# Hygiene promotion might be better than serological screening to deal with Cytomegalovirus infection during pregnancy: a methodological appraisal and decision analysis

**DOI:** 10.1186/s12879-020-05139-8

**Published:** 2020-06-16

**Authors:** Agathe Billette de Villemeur, Pierre Tattevin, Louis-Rachid Salmi, the French Haut Conseil de la santé publique Working Group, S. Alain, D. Antona, Y. Aujard, A. Bégué, T. Barjat, E. Billaud, A. de Villemeur Billette, S. Colson, V. Dufour, D. Jean, J. F. Gehanno, V. des Fontaines Halley, L. Mandelbrot, S. Matheron, P. Minodier, M. Marseille Roussey, D. Royère, L. R. Salmi, O. Scemama, P. Tattevin, F. Teurnier, C. Trastour, C. Vauloup-Fellous

**Affiliations:** 1 Haut Conseil de Santé Publique, F-75000 Paris, France; 2 CHU de Rennes, Service de maladies infectieuses et médecine tropicale, F-35000 Rennes, France; 3 CHU de Bordeaux, Pôle de santé publique, Service d’Information Médicale, F-33000 Bordeaux, France; 4 Univ. Bordeaux, ISPED, Centre INSERM U1219-Bordeaux Population Health, F-33000 Bordeaux, France; 5 INSERM, ISPED, Centre INSERM U1219-Bordeaux Population Health, F-33000 Bordeaux, France

**Keywords:** Cytomegalovirus infection, Pregnancy, Mass screening, Hygiene, Decision support techniques

## Abstract

**Background:**

Cytomegalovirus infection is the most frequent viral congenital infection, with possible consequences such as deafness, or psychomotor retardation. In 2016, the French High Council of Public Health was mandated to update recommendations regarding prevention of cytomegalovirus infection in pregnant women. We summarize a critical appraisal of knowledge and deterministic decision analysis comparing the current no-screening situation to serological screening during pregnancy, and to hygiene promotion.

**Methods:**

Screening was defined as systematic serological testing, during the first trimester, with repeated tests as needed, to all pregnant women. Outcomes were: 1) severe sequela: intellectual deficiency with IQ ≤ 50 or hearing impairment < 70 dB or sight impairment (≤ 3/10 at best eye); 2) moderate sequela: any level of intellectual, hearing or sight deficiency; and 3) death or termination of pregnancy. We simulated the one-year course of cytomegalovirus infection in a cohort of 800,000 pregnant women. We developed a deterministic decision model, using best and min-max estimates, extracted from systematic reviews or original studies.

**Results:**

Relevant data were scarce or imprecise. We estimated that 4352 maternal primary infections would result in 1741 foetal infections, and an unknown number of maternal reinfections would result in 1699 foetal infections. There would be 788 cytomegalovirus-related consequences, including 316 foetal deaths or terminations of pregnancy, and 424 moderate and 48 severe sequelae. Screening would result in a 1.66-fold increase of poor outcomes, mostly related to a 2.93-fold increase in deaths and terminations of pregnancy, not compensated by the decrease in severe symptomatic newborns. The promotion of hygiene would result in a 0.75-fold decrease of poor outcomes, related to both a decrease in severe sequelae among symptomatic newborns (*RR* = 0.75; min-max: 1.00–0.68), and in deaths and terminations of pregnancy (*RR* = 0.75; min-max: 0.97–0.68).

**Conclusions:**

Prevention of cytomegalovirus infection during pregnancy should promote hygiene; serological screening should not be recommended.

## Background

With a prevalence in live births from 0.6 to 6.1% in low-income countries [1] and 0.4 to 0.7% in industrialized countries [2], cytomegalovirus infection is the most frequent viral congenital infection worldwide [3]. Around 87% infected foetuses will not have any sequelae, even among those with severe symptoms at birth [4]; sequelae, however can occur in asymptomatic newborns, and late sequelae can occur up to 7 years after birth [4]. Accurate tools to predict the occurrence and consequences of congenital cytomegalovirus infection are lacking; imaging techniques do not accurately predict prognosis [5, 6]. Although cytomegalovirus infection is the first viral cause of deafness, which is the most frequent sequela [3, 7–11], severe sequelae, such as bilateral deafness, are rare (1–2%), occur in 40% of symptomatic infected newborns [4] and are rarer in asymptomatic infected newborns [4, 12]. The risk of sequelae related to congenital cytomegalovirus infection is similar to that of congenital toxoplasmosis or spina bifida [7, 13].

In the absence of a vaccine against cytomegalovirus [6, 14], some authors have suggested that screening during pregnancy or at birth could be good options to decrease the frequency of poor outcomes [15–17], but the possible benefits of screening has been debated [6, 15, 18–29]. Diagnosis of a primary infection relies on the appearance of IgG, or a significant increase in IgG or presence of IgM; a test of IgG avidity can confirm the date of infection, with an uncertainty of 3 months [30–33]. For optimal screening during pregnancy, tests should ideally be done during the first trimester, because the risk of transmission to the foetus is highest around conception and the performance of tests decreases later during pregnancy [34, 35]. One limit of screening for cytomegalovirus is related to the lack of reliable tests to identify reinfections or reactivations of previously acquired infections [6, 36]. In a population with 50% seroprevalence, the risk of transmission to the foetus and severity of consequences seem similar after reinfections or reactivations than after primary infections [37–43], but the frequency of reinfection remains unknown [6, 44–47].

To our knowledge, no national or international public health authorities have ever recommended screening as a strategy to decrease foetal transmission and its consequences, mostly because there is no effective treatment to propose to infected mothers. Still, some professional organizations have recommended screening during pregnancy or in healthcare professionals in a few countries [6, 18, 21, 22, 28, 48, 49]. Case-finding testing [50] by general practitioners or gynaecologists, as part of routine testing during pregnancy, has also been observed in Belgium, Portugal, Israel and France [15].

In France, two public bodies have considered, in 2002 [51] and 2004 [52], that screening could not be justified, given the absence of an effective treatment. They also argued that the World Health Organisation (WHO) criteria for the implementation of screening programs [53] were not respected. Both recommendations further underscored the need to put more efforts on prevention of cytomegalovirus infection, by focusing on known risk factors, and promoting hygiene [51, 52]. In 2016, the French General Direction of Health (DGS) mandated the French High Council of Public Health (HCPH) to update the latest 2004 recommendations regarding prevention of cytomegalovirus infection in pregnant women.

This paper summarizes the recommendations of the Working Group set by the HCPH to answer the French authorities’ mandate. More specifically, we report the methods and results of a systematic critical appraisal of knowledge regarding cytomegalovirus infection and a deterministic decision analysis which compares the current no-screening situation to two strategies, namely screening during pregnancy and reinforcing hygienic measures, to identify the best strategy to decrease the burden of poor outcomes associated with congenital cytomegalovirus infection.

## Methods

### Scope and general process

The HCPH has constituted a Working Group including a core group of public health specialists, epidemiologists and infectiologists, completed by representatives of stakeholders, including Public Health Agencies, virologists, infectiologists, paediatricians, ethicists, obstetricians, a paediatric nurse, an occupational physician, and a midwife. All members declared they had no potential conflict of interest related to this topic. The Working Group met 14 times to: i) formulate the targeted population, intervention, comparisons and outcomes (PICO) [54], ii) develop the decision model from a representation of the course of the infection, iii) review WHO screening criteria [53] and their adaptation [55], and iv) review the evidence. The Working Group also interviewed other stakeholders, including promotors of screening and patient associations. The last sessions were devoted to discussing conclusions and recommendations, which were approved by a formal vote, following HCPH rules [56]. This report is presented according to a combination of PRISMA for systematic reviews [57] and CHEERS for medico-economic evaluations [58]. The scope of the decision analysis, however, did not cover economic aspects, as there was no clear evidence on the effectiveness of the interventions compared (screening and hygiene promotion) when the work was initiated [59]. The protocol was not registered, but validated by the HCPH.

When building the decision analysis and reviewing evidence regarding screening, the Working Group considered that a recommendation should consider [53, 55]: the public health importance of the problem; the length of the preclinical phase; the reliability and accuracy of tests during the preclinical phase; the availability and effectiveness of a treatment during the preclinical phase; the risk-effectiveness balance associated with systematic serological screening.

### Definition of compared interventions

We compared the current French situation, including one visit each month, at least four serology tests (toxoplasmosis; rubella; syphilis; hepatitis B virus) between 10 and 15 weeks of amenorrhea, and three echography exams around 9–11, 20–25 and 30–35 weeks of amenorrhea [60], with two strategies that would either introduce cytomegalovirus screening during pregnancy, or promote hygiene. The current situation was defined as recommended in 2004, i.e. no cytomegalovirus screening [52], neither during pregnancy nor at birth; there is however, since 2014, a national program promoting screening of hearing deficiency at birth [61]. The screening strategy would offer all pregnant women a systematic cytomegalovirus serology, during the first trimester, with possible repeated tests as needed. The hygiene strategy would consist in reinforcing hygiene measures through a strong and repeated promotion among pregnant women, the public and health professionals, as previously shown as effective in several countries [17, 62–64]. Specific modalities were not defined, but we assumed that hygiene would be applied vigilantly [65].

### Search strategy and selection criteria

Literature review started by identifying references assessed in the 2002 and 2004 reports [51, 52], and reviews published since [1, 2, 4–7, 10, 12, 14–16, 19, 23, 25, 29, 30, 34, 39, 49, 66–96]. Then, each member of the Working Group provided the literature regarding the topic they were in charge of. Relevant references were sought in Pubmed/Medline, Cochrane database, Google Scholar, and “banque de données en santé publique”, until 2017. Inclusion criteria covered articles published in French or English since 2002, completed with the evidence covered in the previous recommendations. Keywords or free-text expressions used were “congenital cytomegalovirus infection”, “congenital infection”, “TORCH”, “cytomegalovirus”, and “stillbirth”, “mortality”, “case fatality”, “termination of pregnancy”, “miscarriage”, “sensory neuro hearing loss”; complementary searches also included “transmission”, “vertical transmission”, “immunity”, “immune defence”, “day care centres”, “variability”, “contamination route”, “primary infection”, “reactivation-reinfection”, “recommendations”, “program”, “pregnancy”, “foetus”, “newborns”, “prevention”, “epidemiology”, “prevalence”, “incidence”, “symptomatology”, ‘low-birth weight”, “small size for gestational age”, “prognosis”, “follow up”, “outcome”, “sequelae”, “microcephaly”, “mental deficiency”, “mental disorder”, “visual disorder”, “sensorineural hearing loss”, “autism”, “screening”, “testing”, “assay”, “serology”, “diagnosis”, “predictive value”, “sensitivity and specificity”, “diagnostic accuracy”, “avidity”, “PCR”, “hygiene”. Whenever identified from reference lists of previously selected articles, articles and guidelines in other languages (Portuguese, German, and Hebrew) were translated. The search started with systematic reviews and meta-analyses, but all articles based on randomized controlled trials, case-control studies or other observational studies were used as needed, including opinion papers, to identify potential relevant evidence. We also completed our search by interviewing experts, and reading conferences abstracts. Data were also asked from Public Health agencies (Santé Publique France; Agence de la Biomédecine), registries of children with handicaps, National Reference Centres for the control of transmissible diseases and Pluridisciplinary Centres for Prenatal Diagnostic. Level of evidence was graded using SIGN checklists (available at https://www.sign.ac.uk/checklists-and-notes.html; accessed February 24, 2020).

### Construction and analysis of decision model

Outcomes were defined as follows: 1) severe sequelae: intellectual deficiency with Intelligence Quotient (IQ) ≤ 50 or severe hearing impairment < 70 dB or severe visual impairment (≤ 3/10 for the best eye); 2) moderate sequelae: any level of intellectual deficiency, hearing or sight impairment; and 3) death or termination of pregnancy.

We simulated the course of cytomegalovirus infection in a virtual cohort of 800,000 pregnant women, which is the estimated number of pregnancies in France in 2010, based on the number of live births. The time horizon was 1 year.

All parameters were extracted, wherever available, either from meta-analyses, or other systematic reviews, observational studies based on representative samples, prospective or historical cohorts or randomized trials. Studies with recruitment bias, major losses to follow up, or poor case definitions were used only if a parameter could not be found elsewhere. Case reports or case series were excluded. As no single study adequately described the course of the infection, from a healthy seronegative woman to the observation of sequelae in children, we used data from studies describing one stage of the course of the infection. Probability of an event at a given stage was multiplied by the probability of the next event.

We developed a deterministic decision model, using best and min-max estimates (Table 1). Whenever the literature provided several estimates for a given parameter, we used the mean of available values as best estimate. For min-max models, we used the lowest and highest limits of reported confidence intervals, or the minimum and maximum of all available estimates. When the expert group considered that an extreme value was either not coherent with the French context or considered unrealistic or incompatible with calculation (for instance a test specificity of 100%), we used minimum or maximum point estimates reported in a meta-analysis.

**Table 1:** Data sources and parameters regarding cytomegalovirus infection during pregnancy, potential screening tests and hygiene measures

Parameter	Sources	Best estimate	Min-Max	Comments
Sero-prevalence in 15–49 years-old women	[97]	45.6%	25.2–61.0%	Robust French representative survey
Incidence of MPI		1.0%	0.2–1.4%	Mean incidence and mean of CI lower and higher limits (expert consensus)
Transmission rate from mother to foetus	[19, 36, 53, 84, 87, 98–100]	40.0%	5.0–72.2%	Mean transmission rates and CI lower and higher limits from studies reporting rates by trimester of pregnancy
Transmission rate from mother to foetus (1st trimester)	[39, 88, 98, 101]	19%	NA	Mean incidence (expert consensus); screening scenario only
Transmission rate from mother to foetus (2nd trimester)	[39, 88, 98, 101]	36%	NA	Mean incidence (expert consensus); screening scenario only
Transmission rate after reinfection^b^	[42, 102–104]			Unknown; assumed equal to transmission after MPI
Proportion of infected newborns who are symptom free	[2, 4, 105, 106]	87.3%	Min: 75.0%	Stable across studies
Proportion of medical TOP among MPI or infected foetus	[2, 4, 20, 23, 25, 39, 41, 43, 74, 88, 89, 105, 107–120]	9.2%	NA	Data from National Reference Laboratory and literature
Proportion of medical TOP after screening	[85, 103]	95.0%	NA	Foetal infections confirmed by amniocentesis, positive or not at echography
Prevalence of infection at birth	[1, 2, 4, 38, 42, 68, 69, 97, 98, 112, 121]	0.43%	0.20–0.61%	Min-max from European studies, vary with selection and tests
Proportion of infected newborns who are symptomatic	[2, 4, 88, 105, 106]	12.7%	NA	Do not include TOP, part of whom would have died^c^
Proportion of infected newborns who are symptomatic born from mothers with immunity prior to pregnancy	[38, 39, 42, 105, 122]	12.7%	NA	Stable across studies
Incidence of hearing impairment between birth and 5 years among asymptomatic newborns with sequelae	[9]	53.0%	NA	
Frequency of any sequelae in asymptomatic newborns	[4, 11, 12, 43, 98, 106, 123, 124]	13.3%	NA	
Frequency of any severe sequelae in severe symptomatic newborns	[4, 9, 10, 19, 42, 43, 162, 163, 173]	47.0%	NA	Middle of value range
Frequency of any moderate sequela in severe symptomatic newborns	[4, 9, 19, 42, 43, 105, 106, 124]	25.0%	NA	Middle of value range
Frequency of any moderate sequela in moderately symptomatic newborns	[4, 9, 19, 42, 43, 105, 106, 124]	16.0%	NA	Middle of value range
Proportion of any severe symptomatic newborns without sequela	[105]	28.0%	NA	Middle of value range
Proportion of any moderately symptomatic newborns without sequela	[105]	51.0%	NA	Middle of value range
Frequency of any severe sequelae in moderately symptomatic newborns	[105]	33.0%	NA	Middle of value range
Proportion of any late sequelae among symptomatic newborns with sequelae	[105, 106, 125]	43.0%	NA	
Sensitivity IgG	[126–128]	99.7%		Diasorin test; false negative women considered negatives, but MPI and consequences considered in truly infected women
Specificity IgG	[126–128]	99.4%		Abbott test; false positive women considered positives, but MPI and consequences considered in truly infected women
Sensitivity IgM	[128–131]	94.0%	79.4–95.9%^a^	Vidas, Beckman-Coulter, Diasorin, Roche, Siemen HC tests
Specificity IgM	[128–132]	99.3%	96.4–100%^a^	
Sensitivity avidity of IgG	[23, 34, 36, 109, 131, 133–142]	83,0%		During first 12 weeks of pregnancy; applied when IgM positive
Specificity avidity of IgG	[23, 34, 133–143]	82,0%		During first 12 weeks of pregnancy; applied when IgM positive
Absolute reduction with hygiene	[63]	−50%		Group consensus on most plausible result

*MPI* maternal primary infection; *TOP* termination of pregnancy; ^a^ Maximum values are point estimates from studies not providing confidence intervals; ^b^, unknown, considered equal to previous line; NA: min-max not considered in robustness analyses; ^c^ Min = 0 from [4] disregarded by group as not plausible

Because some key data were lacking, we made the following choices or hypotheses. 1) Prevalence of maternal Cytomegalovirus infection was taken from a French representative survey [97], rather than from a meta-analysis including non-representative studies [2]. Because this prevalence also varied dramatically across countries and French regions, we used age-specific prevalence to compute the minimum and maximum prevalence. 2) Because the number of reinfections or reactivations in women with preconception immunity is unknown [46, 144, 145], we hypothesized that the number of newborns infected would be the same in women with preconception immunity, after a reinfection or reactivation, and after a primary infection, in line with literature data [2, 6, 19, 33, 37–39, 42, 43, 87, 88, 102, 144–146]. 3) To estimate the potential impact of cytomegalovirus serological screening, we applied sensitivity and specificity estimates for the main tests used in France. 4) To consider the fact that infections occurring just before a pregnancy can have consequences for the foetus [39, 88, 98, 101], varying transmission rates by pregnancy trimesters, and the fact that seroconversion late during pregnancy would not leave enough time to carry all exams, and the relatively moderate or low severity of late infections, we estimated the overall rate by dividing the time of transmission in four trimesters (prior to conception, and three pregnancy trimesters), and hypothesized that no intervention would be done during the last trimester. 5) To estimate the potential impact of hygiene, we used a conservative rate reduction found in a French study [64], considering that studies carried elsewhere lacked a control group and thus were overoptimistic and unrepresentative of the compliance expected in France.

## Results

### Decision model

The PICO and decision models were formulated from a public health perspective, to assess whether screening during pregnancy (intervention 1) or promotion of hygiene through information campaigns targeting the public and healthcare professionals (intervention 2) would decrease the frequency of children infected by cytomegalovirus and having sequelae, decrease the frequency of infected foetuses resulting in termination of pregnancy, and decrease the number of deaths in newborns and toddlers (outcomes), compared to care usually provided, which does not include screening (comparator).

### Data source

**Fig. 1:**
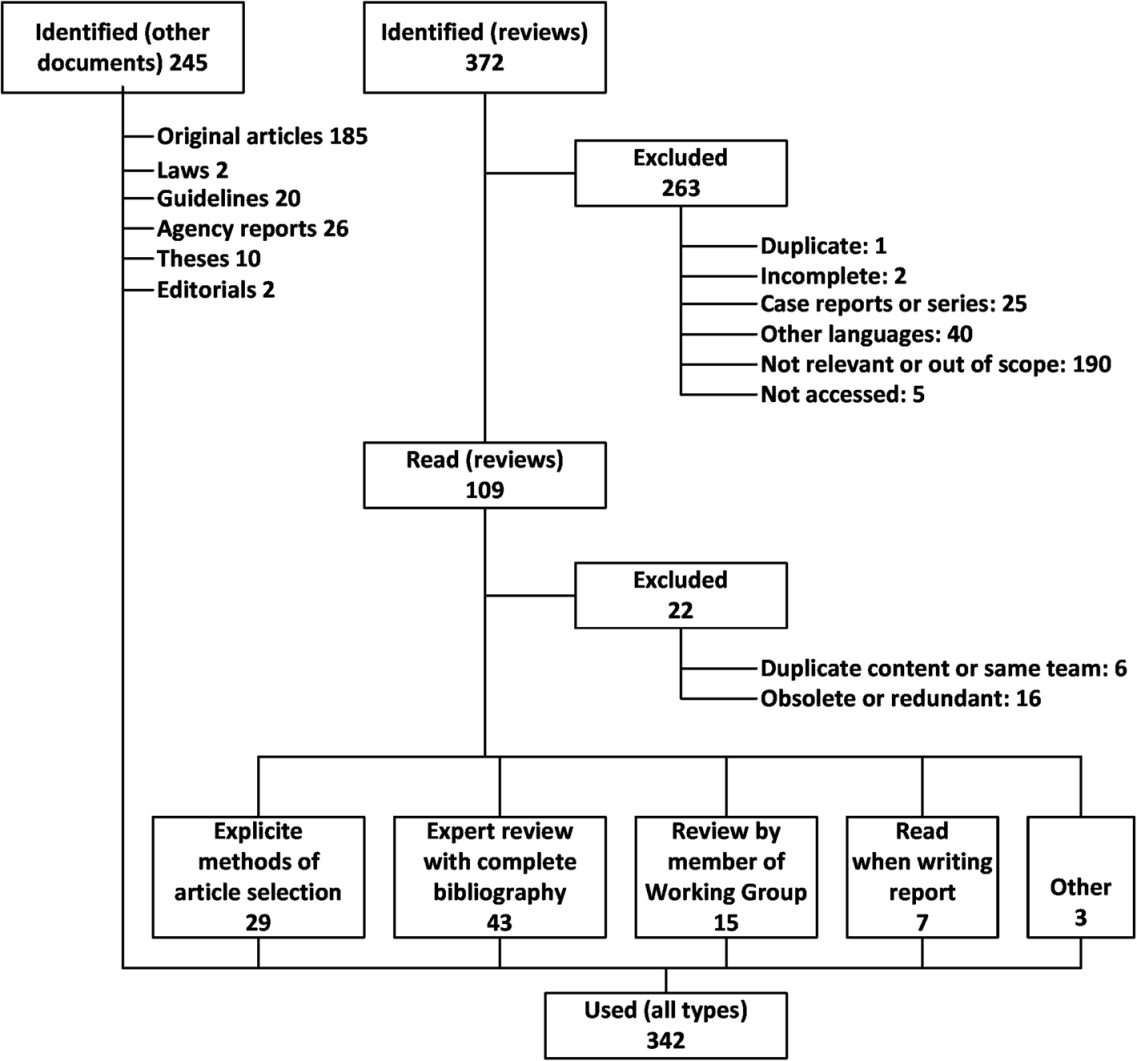
PRISMA flow chart

The Working Group reviewed 572 references, including 90 systematic reviews (Fig. 1). In general, data were scarce and often very imprecise (Table 1). Min-max estimates were used in the model only for sero-prevalence, incidence of maternal primary infection, transmission rate from mother to foetus, prevalence of infection at birth, and sensitivity and specificity of IgM tests; for the proportion of infected newborns free of symptoms, we only used the best and minimal estimate. For the screening scenario, the transmission rate from mother to foetus had to be estimated separately, depending on the time of transmission, as the rate during the first and second trimester are different, and a transmission during the third trimester was deemed too late to allow any early intervention. Best estimates for these transmission rates were based on expert consensus, as were the estimates for the effectiveness of hygiene promotion.

### Course of cytomegalovirus infection during pregnancy

**Fig. 2:**
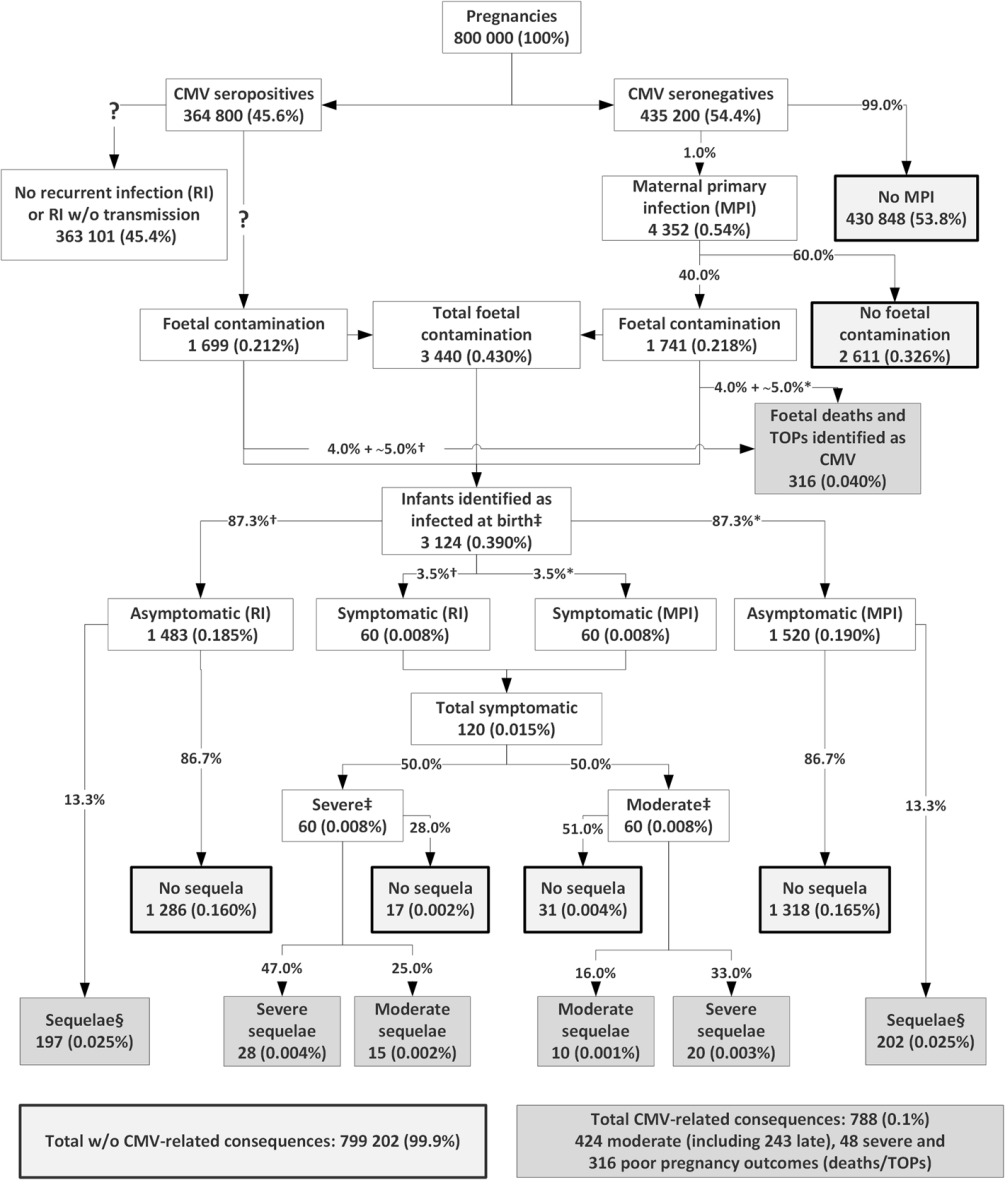
Course of cytomegalovirus infection during pregnancy in the current French situation where screening is not recommended. Dark grey boxes correspond to poor outcomes; light grey boxes with a bold outline correspond to favourable outcomes; CMV: cytomegalovirus; RI: recurrent infection; MPI: maternal primary infection; TOP: termination of pregnancy; w/o: without; *Among MPI-related foetal infections (total = 100% when including medical abortions and foetal deaths); †Among RI-related foetal infections (total = 100% when including medical abortions and foetal deaths); ‡Data unavailable to identify whether RI or MPI; § Usually moderate, exceptionally severe

In France, for a typical cohort of 800,000 pregnancies, we estimated there would be 4352 maternal primary infection, that would result in 1741 foetuses being affected by cytomegalovirus and an unknown number of maternal reinfections, that would result in 1699 foetus being affected by cytomegalovirus (Fig. 2). These foetal infections would result in a total of 788 cytomegalovirus-related consequences, including 316 foetal deaths or terminations of pregnancy, 424 moderate sequelae, and 48 severe sequelae.

### Potential impact of systematic serological screening and hygiene promotion

Compared to the current French situation, with the introduction of IgG, IgM and avidity of IgG in negative in the first trimester and, in the second trimester, of IgG for women previously negative, serological screening would correctly identify 2780 MPIs and result in 484 false negatives and 238 false positives, and a total of 3018 women would be considered MPIs. Screening would result in a 1.66-fold increase (min: 1.13; max: 2.16) of poor outcomes, from 788/800000 to 1307/800000 (Table 2). This increase would be mostly related to a 2.93-fold increase (min: 1.9; max: 4.38) in deaths and terminations of pregnancy, which would not be outbalanced by a decrease in severe symptomatic newborns (*RR* = 0.83; min-max: 0.96–0.71) and severe sequelae in symptomatic newborns (Relative Risk (*RR*) = 0.83; min-max: 1.00–0.71).

**Table 2:** Cytomegalovirus infection during pregnancy, and potential impact of screening or a prevention program promoting hygiene. Figures are numbers (%) for the best and minimal and maximum; % are provided with one decimal when > 1%, two decimals when ≤1% but > 0.1%, and three decimals when ≤0.1%.

State	Current situation			Screening			Promotion of hygiene		
	Best (%)	Minimum (%)	Maximum (%)	Best (%)	Minimum (%	Maximum (%)	Best (%)	Minimum (%	Maximum (%)
CMV seronegative	435,200 (54.4)	598,400 (74.8)	312,000 (39.0)	433,683 (54.2)	595,414 (74,4)	310,128 (38,8)	435,200 (54.4)	598,400 (74.8)	312,000 (39.0)
MPI	4352 (0.54)	1197 (0.15)	4368 (0.55)	3018 (0.38)	1270 (0.16)	3069 (0.38)	2176 (0.27)	598 (0.07)	2184 (0.27)
Foetal infection in CMV-	1741 (0.22)	60 (0.0008)	3154 (0.39)	1741 (0.22)	60 (0.008)	3154 (0.39)	870 (0.11)	30 (0.004)	1577 (0.20)
CMV seropositive	364,800 (49.6)	201,600 (25.2)	488,000 (61.0)	366,317 (45.8)	204,586 (25.6)	488,408 (61,1)	364,800 (45.6)	201,600 (25.2)	488,000 (61.0)
Foetal infection after RI	1699 (0.21)	1540 (0.19)	1726 (0.22)	1699 (0.21)	1540 (0.19)	1726 (0.22)	1699 (0.21)	1540 (0.19)	1726 (0.22)
Total foetal infections	3440 (0.43)	1600 (0.20)	4880 (0.61)	3440 (0.43)	1600 (0.20)	4880 (0.61)	2569 (0.32)	1570 (0.20)	3303 (0.41)
Deaths & TOP	316 0.040)	147 (0.018)	449 (0.056)	919 (0.12)	183 (0.023)	1968 (0.25)	236 (0.030)	144 (0.018)	304 (0.038)
Total congenital infections	3123 (0.39)	1453 (0.18)	4431 (0.55)	2522 (0.32)	1417 (0.18)	2913 (0.36)	2333 (0.29)	1425 (0.18)	2999 (0.37)
Symptomatic in RI	60 (0.008)	54 (0.007)	60 (0.008)	60 (0.008)	54 (0.007)	60 (0.008)	60 (0.008)	54 (0.007)	60 (0.008)
Symptomatic in MPI	60 (0.008)	2 (< 0.001)	110 (0.014)	41 (0.005)	1 (< 0.001)	61 (0.008)	30 (0.004)	1 (< 0.001)	55 (0.007)
Severe symptomatic	60 (0.008)	28 (0.004)	85 (0.011)	50 (0.006)	27 (0,003)	60 (0.008)	45 (0.006)	27 (0,003)	58 (0.007)
Moderate symptomatic	60 (0.008)	28 (0.004)	85 (0.011)	50 (0.006)	27 (0,003)	60 (0.008)	45 (0.006)	27 (0,003)	58 (0.007)
Sequelae asymptom. RI	197 (0.025)	179 (0.022)	200 (0.025)	197 (0.025)	179 (0.022)	200 (0.025)	197 (0.025)	179 (0.022)	200 (0.025)
Sequelae asymptom. MPI	202 (0.025)	7 (< 0.001)	366 (0.05)	125 (0.016)	2 (< 0.001)	171 (0,022)	101 (0.013)	3 (< 0.001)	183 (0.023)
Severe sequela (symptom)	48 (0.006)	22 (0.003)	68 (0.009)	40 (0.005)	22 (0.003)	48 (0.006)	36 (0.005)	22 (0.003)	46 (0.006)
Mod. sequelae (symptom)	35 (0.004)	11 (0.001)	35 (0.004)	21 (0.003)	11 (0.001)	25 (0.003)	18 (0.002)	11 (0.001)	24 (0.003)
Total poor outcomes	788 (0.099)	367 (0.046)	1119 (0.14)	1301 (0.16)	397 (0.050)	2413 (0.30)	588 (0.074)	356 (0.045)	757 (0.095)

*CMV* cytomegalovirus; *RI* recurrent infection in CMV seropositive mothers; *MPI* maternal primary infection; *CMV-* mothers who are CMV seronegative before pregnancy; *TOP* termination of pregnancy; asymptom.: if asymptomatic at birth; *Mod.*: moderate; symptom: if symptomatic at birth

Compared to the current French situation, the promotion of hygiene would result in a 0.75-fold decrease (min: 0.97; max: 0.68) of poor outcomes, from 788/800000 to 588/800000 (Table 2). This would be related to both a decrease in severe sequelae among symptomatic newborns (*RR* = 0.75; min-max: 1.00–0.68), and in deaths and terminations of pregnancy (*RR* = 0.75; min-max: 0.97–0.68).

## Discussion

### Main findings

To our knowledge, this is the first attempt to compare promotion of hygiene and systematic serological screening as interventions to deal with cytomegalovirus infection during pregnancy. Our review of the evidence and model suggest that screening of cytomegalovirus infection during pregnancy would actually increase the risk of poor outcomes. Compared to the current French situation, promotion of hygiene would result, each year, in 12 less children with severe sequelae, around a hundred less children with moderate sequelae, and would avoid a quarter of cytomegalovirus-related foetal deaths and medical terminations of pregnancy.

The main limit of modelling the effect of screening during pregnancy on the course of cytomegalovirus infection is the absence of a treatment with proven effectiveness in this context. Thus, if cytomegalovirus infection is diagnosed during pregnancy, the only interventions to consider are termination of pregnancy and potentially harmful antiviral or immunoglobulin treatments, with unproven effectiveness [5, 79, 83, 86]. The only published evidence that valancyclovir might be effective came from an uncontrolled trial [107], and no study, to our knowledge, has addressed side effects of available treatments, without any robust data on the tolerability of such regimen during pregnancy. Further, our model is based on available evidence which was often of low quality. Some authors have suggested that with serological tests to accurately date the maternal infection and safe foetal tests to accurately predict the occurrence of sequelae, screening could help better advise parents, who would, as autonomous adults, decide whether to terminate pregnancy or not [30, 147–149]. These ideal testing and prognostic conditions are currently unlikely to occur in any healthcare system [80]. Notably, most criteria set by the WHO to justify screening programs cannot be documented by appropriate evidence regarding 1) the availability and effectiveness of treatments [6, 150]; 2) the actual magnitude of all dimensions of the problem, especially in women who are already seropositive and in children in the long term; 3) the reliability and validity of screening tests in a context of early infection and low prevalence; and 4) the lack of easily applicable prognostic markers to define women, foetuses, and children at risk of developing poor outcomes [6, 150]. Given that echography and magnetic resonance imaging still have numerous false negatives [77, 80], follow up and prognosis could be based on amniocentesis. An amniocentesis can confirm that a foetus is infected, and the likelihood of sequelae after a false negative is very low [151]. However, the predictive value of amniocentesis findings is poorly documented, as no follow-up study included systematic autopsy [103].

### Strengths and limitations

One major limit, as was noted in previous systematic reviews [1, 2, 4–7, 10, 12, 14–16, 19, 23, 25, 29, 30, 34, 39, 49, 66–96], is the lack of high-grade evidence. No cohort study describes the full course of cytomegalovirus infection, from women of childbearing age, through conception, pregnancy, birth, to long-term follow up of children with sequelae. The only available cohort studies focused on one or a few steps of the course of the disease, providing only partial data [149]. Moreover, large studies are scarce, and a full cohort would require, given that cytomegalovirus congenital infection is rare, a huge number of women, which is probably not feasible.

Another limit is that our modelling of the course of the infection and the impact of hygiene promotion was based on average transmission frequencies. It has been shown that the frequency of MPIs increased from 5% around conception to 70% in the third trimester [88, 98, 101]. Because the frequency of transmission following reinfection remains undocumented throughout pregnancy, and the severity of infection decreases with late transmission, we believe the use of average transmission frequency provides a reasonable estimate of poor outcome frequency. Also, limiting the screening model to the impact on first-trimester transmissions and resulting outcome would not affect the overall result of the comparisons. Estimates of the potential impact of hygiene only came from studies conducted among women aware of their serological status; nevertheless, as it was shown that the main determinant of adherence to hygiene was the fact of being pregnant [152], we believe our estimated impact of hygiene is reasonable. Ideally, however, we would need confirmation of this effect in cohorts of pregnant women who are unaware of their serological status. Similarly, in the absence of studies focusing on the impact of hygiene on reinfections in seropositive women, we have not considered in our model the possibility of such an impact. Therefore, if messages promoting hygiene are well framed, the effects might even be larger than estimated, as already suggested for toxoplasmosis [65].

Another limit of the literature is the heterogeneity of the elements used by authors to define cases, regarding 1) number and types of symptoms considered at birth (clinical definition, including or not hypotrophy…) [2, 4, 9, 12, 81, 86]; 2) types of imaging or other tests used [5, 6, 29, 108, 147, 150, 153–161]; and 3) classification of intermediary avidity results (considered to be linked either with recent or past infection in different studies) [23, 24, 109, 162]. Losses to follow up were seldom considered, and many studies did not report foetal deaths in utero, stillbirths, or terminations of pregnancy [110]. No randomized trial ever evaluated screening; some observational studies did not include a comparison group, or only drew comparison with historical cohorts, some of which seem outdated [85]. Most studies of foetal death or post-neonatal fatalities did not include autopsies; the interpretation of autopsy findings is questionable, as there is no clear correlation between lesions found in cytomegalovirus-infected foetuses and the occurrence of sequelae [111, 147, 163, 164]. In addition, many comparative studies did not adjust for key confounding factors such as age, parity, occupation, or risk factors for infection.

Consequently, we sometimes had to use imprecise estimates and strong hypotheses. Still, the estimated number of severe sequelae for the course of infection is consistent with the numbers observed locally by handicap registries, extrapolated to France, and with the results of a comprehensive survey [112], even though these estimates might be underestimated because a cytomegalovirus cause can be missed as tests based on the dried blood spots have a low sensitivity [30, 165]. We still believe our estimates of severe sequelae frequency are accurate enough to estimate the impact of screening, as screening would only detect MPIs [91]. We also modelled the course of disease and the potential impact of screening and hygiene using an incidence of MPI of 1%, as lower values reported in France [64] and in the Netherlands [166] were considered unrealistic by the Working group or likely linked to contexts where hygiene was much better than usual practices. One study reported much higher estimates, but was clearly overestimating the incidence of sequelae in infants, because the results of intermediate calculations were inappropriately rounded [19]. Another hypothesis was that the risk of foetal infection would be the same, whether women were already seropositive or not. Suggestions of higher risk following reinfections came from non-comparative case series of seropositive women [5, 68, 69, 72], or from studies where the risk of transmission was poorly documented in seropositive women [5, 72]. One of the strongest hypotheses concerns the frequency of pregnancy terminations related to the increased positive detection following screening [98, 103, 155]. This hypothesis, however, is coherent with European data, suggesting that pregnancy termination is more likely to be proposed than the option of welcoming a handicapped child [113]. One strength of the models is that we used a specific definition of moderate to severe sequelae. Some authors have suggested that intellectual deficiency can be observed in children with sensory neuro hearing loss, but this broader definition of possible sequelae came from non-comparative studies [4, 123, 167], and this disappeared in comparative studies, where asymptomatic newborns who have only an SNHL never have intellectual deficiency [12, 43]. Therefore, more evidence is clearly needed regarding the effectiveness of behavioural interventions to promote hygiene, the frequency of reinfection, and the information given to parents to make decisions, especially in relation to TOPs. Appropriate randomized controlled trial must also assess the effect of treatments, including on the severity of sequelae.

### Interpretation

To our knowledge, screening is not recommended by any national public health institution. Nevertheless, during interviews carried out by the Working Group, we identified practices of systematic prenatal screening at the level of one or several maternities, in France and in Israel [24, 110]. In the latter country, this practice is associated with up to 50% voluntary or medical terminations of pregnancy [24, 110]. In Canada, a screening can be proposed to professionals who work with young children [49]; the same recommendation exists in Portugal but is poorly applied [28]. Beyond the results of our simulation, not recommending eviction from work (as applied for instance in Belgium [22, 48]) and screening in France is also justified by two facts [168]: 1) prevalence of infection is slightly higher in professionals than in families [78, 93, 168–171], though the difference disappears when hygienic measures are applied in professionals [170, 172–177]; and 2) when professionals are at home, they tend not to apply hygienic measures as consistently [7, 85, 168].

Until randomised trials demonstrate that a treatment is safe and effective to deal with cytomegalovirus congenital infection, the best strategy seems to be hygiene promotion, an educational intervention that would be relatively inexpensive and poses essentially no risk. Nevertheless, effective treatment should only be considered as a last resort, if infection occurs, and reinforcement of hygiene should always be promoted. Although the general principles of these measures are well known [6, 62, 84], we did not specify the nature of the promotion tools and organization. Hygiene measures are meant to decrease contact with urine, saliva, nasal and lachrymal fluid of young children [71]. They include handwashing and recommendations for young women, pregnant or with a project of pregnancy, and their partner to avoid sucking their child’s spoons or teats, finishing their child’s meals, sharing their toilet utensils, and kissing the face of a child who cries. Use of a condom is also recommended with a new or casual sex partner or when the partner is likely to be infected with cytomegalovirus [71]. Although some of these measures seem difficult to adopt in cultures where cuddling and consoling toddlers is usual, we found several studies documenting their effectiveness [25, 63, 178]. Our simulation, however, used a conservative estimate of halving MPI risk [25] whereas other studies that focused only on MPIs found reductions around 85% in that group [25, 63, 178]. These studies, however, were not randomized [84], compared with a non-comparable historical period [64] or another maternity where no information was provided [63].

The effectiveness could thus even be higher than simulated here, if recommendations were made to all women, regardless of the serology status, as hygiene would decrease both MPIs and reinfections [6, 73, 152, 179]. There are also too many uncertainties regarding the frequency of reinfections; studies dramatically fail to consider the raising anxiety related to screening, information on risk, stigmatization and the anxiety of parents who could have an infected child with sequelae [65, 180–182], especially if they have applied rigorous hygiene measures.

Professional and public health bodies should promote a better knowledge regarding cytomegalovirus in professionals and women. Knowledge regarding cytomegalovirus congenital infection is indeed insufficient in France and many other countries [6, 62, 65, 179, 181, 183–189]. The proportion of pregnant women who say they know about cytomegalovirus vary from 12.5 to 39.0% across countries [188]; this proportion goes up to 55.7 to 74.0% where reinforced information is associated to serology [184, 188], but this increase is more related to knowing that one is pregnant than to knowing the results of the serology [65, 178]. Moreover, women who are seropositive are likely to stop respecting hygiene measure consistently [65, 81], and are usually not followed as there is no test to identify reinfections outside of research projects [5, 36]. In most countries, cytomegalovirus is less known than diseases such as toxoplasmosis, human immunodeficiency virus, hepatitis B virus, rubella, autism, syphilis, sudden infant deaths, B streptococcus, Down syndrome, foetal alcohol syndrome, spina bifida, listeria, or parvovirus B19 [183–185, 187, 188]. One obstacle to an appropriate information of pregnant women, however, is that health professionals themselves have a poor knowledge regarding the modes of transmission, maternal symptoms, neonatal complications and effective preventive measures [180, 190–192].

## Conclusions

This review of the impact of hygiene promotion and systematic serological screening, as interventions to deal with cytomegalovirus infection during pregnancy, suggests that systematic screening would increase the risk of poor outcomes. Until randomised trials demonstrate that a treatment is safe and effective to deal with cytomegalovirus congenital infection, prevention of cytomegalovirus infection during pregnancy should primarily promote hygiene reinforcement. Serological screening should not be recommended.

## Data Availability

The datasets used and/or analysed during the current study are available from the corresponding author on reasonable request.

## Abbreviations

CHEERS: Consolidated Health Economic Evaluation Reporting Standards
CMV: Cytomegalovirus
DGS: French General Direction of Health
HCPH: French High Council of Public Health
IQ: Intelligence Quotient
MPI: Maternal Primary Infection
PICO: Population Intervention Comparisons Outcome
PRISMA: Preferred Reporting Items for Systematic Reviews and Meta-analyses
RI: Recurrent Infection
RR: Relative Risk
SIGN: Scottish Intercollegiate Guidelines Network
TOP: Termination of Pregnancy
TORCH: TOxoplasmosis Rubella Cytomegalovirus Herpes
WHO: World Health Organizations
